# Dolphins Stranded along the Tuscan Coastline (Central Italy) of the “Pelagos Sanctuary”: A Parasitological Investigation

**DOI:** 10.3390/pathogens9080612

**Published:** 2020-07-27

**Authors:** Giuliana Terracciano, Gianluca Fichi, Antonia Comentale, Enrica Ricci, Cecilia Mancusi, Stefania Perrucci

**Affiliations:** 1 Istituto Zooprofilattico Sperimentale del Lazio e della Toscana “M. Aleandri”., S. dell’Abetone e del Brennero n. 4, 56123 Pisa and Viale Europa n. 30, 58100 Grosseto, Italy; giuliana.terracciano@izslt.it (G.T.); enrica.ricci@izslt.it (E.R.); 2 Dipartimento di Scienze Veterinarie, Università di Pisa, Viale delle Piagge n.2, 56124 Pisa, Italy; a.comentale@hotmail.it; 3 ARPAT, Agenzia Regionale per la Protezione Ambientale Toscana, Via Marrani 114, 57126 Livorno, Italy; c.mancusi@arpat.toscana.it

**Keywords:** parasites, *Stenella coeruleoalba*, *Tursiops truncatus*, *Grampus griseus*, Tuscany (central Italy), Pelagos Sanctuary

## Abstract

Parasite monitoring is considered a necessary step for cetacean management and conservation. Between February 2013 and July 2015, 26 dolphins (15 *Stenella coeruleoalba*, 10 *Tursiops truncatus*, and one *Grampus griseus*) stranded along the Tuscan coastline of the protected marine area “Pelagos Sanctuary”, were examined. Organs, tissues, and faecal and blood samples taken from all animals were analysed by parasitological, immunological, and molecular techniques. Twenty-one out of 26 dolphins (80.77%) tested positive for at least one parasite species, and 13/15 (86.7%) *S. coeruleoalba*, 7/10 (70%) *T. truncatus*, and the single *G. griseus* were found positive. Identified parasites included the nematodes *Skrjabinalius guevarai* (7.69%, 2/26), *Halocercus lagenorhynchi* (3.85%, 1/26), *Halocercus delphini* (7.69%, 2/26), *Stenurus ovatus* (7.69%, 2/26), *Crassicauda* spp. (7.69%, 2/26); the trematodes *Pholeter gastrophilus* (26.92%, 7/26), *Campula palliata* (3.85%, 1/26); the cestodes *Phyllobothrium delphini* (42.31%, 11/26), *Monorygma grimaldii* (23.08%, 6/26), *Tetrabothrium forsteri* (7.69%, 2/26), *Strobilocephalus triangularis* (7.69%, 2/26), and the acanthocephalan *Bolbosoma vasculosum* (7.69%, 2/26). Moreover, 6/26 (23%) animals scored positive to *Toxoplasma gondii* at serology, but PCR confirmed the infection (*T. gondii* Type II genotype) in a single animal. In examined dolphins, obtained results showed a high prevalence of endoparasites, which included species considered as a cause of severe debilitation or death.

## 1. Introduction

The International Sanctuary for the Protection of Mediterranean Marine Mammals (hereafter Pelagos Sanctuary) is the first International high seas marine protected area worldwide, and it has been added in the list of specially protected areas of Mediterranean interest [1]. The Pelagos Sanctuary encompasses over 87,500 km^2^ of the north-western Mediterranean Sea. It is bounded to the west by a line extending from the Escampobariou Point (N 43°01′70–E 06°05′90), Giens Peninsula, France to the Falcone Cape (N 40°58′00–E 08°12′00), Sardinia, Italy and to the east by another line extending from the Ferro Cape (N 41°09′18–E 09°31′18), Sardinia, Italy to Fosso Chiarone (N 42°21′24–E 11°31′00), Tuscany, Italy [2,3].

The Sanctuary includes the Ligurian Sea and parts of the Corsican and Tyrrhenian seas, and it is composed by the internal maritime (15% of its extent) and territorial waters (32%) of France, Monaco, and Italy, as well as the adjacent high seas (53%) [2,4]. The Pelagos Sanctuary contains habitat suitable for the breeding and feeding needs of the entire complement of cetacean species regularly found in the Mediterranean Sea [2].

The three most abundant cetaceans in the Pelagos Sanctuary are the fin whale, *Balaenoptera physalus* (Lacépède, 1804), the striped dolphin, *Stenella coeruleoalba* (Meyen, 1833), and the bottlenose dolphin, *Tursiops truncatus* (Montagu, 1821). However, five other species are regular components of the Sanctuary’s cetacean fauna: sperm whale, *Physeter macrocephalus;* long-finned pilot whale *Globicephala melas* (Traill, 1809); Risso’s dolphin, *Grampus griseus* (Cuvier, 1812); common dolphins, *Delphinus delphis*; and Cuvier’s beaked whale, *Ziphius cavirostris* (Cuvier 1823) [2,4].

Habitat degradation, interaction with fisheries, and climate change are considered important issues interfering with the conservation of cetaceans worldwide [5,6,7]. Indeed, in the last decades the frequency of cetacean unusual mortality and stranding events has increased worldwide, including in the Mediterranean Sea [8,9,10,11].

Although the role of parasitic diseases as factors in cetacean stranding behaviour is still a topic of current debate, according to some authors, parasites should be included among the potential causes of the cetacean debilitation and death [12]. The damage and mortality of individuals and populations caused by parasitic infections are dependent upon several factors, including the parasite species, its abundance, and the health status of the host [12]. The knowledge of pathological effects and importance of parasites in ecological and evolutionary studies of cetaceans is highlighted, as parasites can influence the behaviour and population size of their hosts and the dynamics of the food chain and community structure [13]. Therefore, information about parasite infections is considered a necessary step towards assessing the impact of parasites on the marine mammal ecology and health and, ultimately, the cetacean population size, and towards the adoption of effective management and conservation plans [14,15].

With the aim to give a contribution to the knowledge of the parasite fauna of stranded cetaceans, this study reports data about parasite species identified in 26 dolphins stranded along the Tuscan coasts of the Pelagos Sanctuary in the period between February 2013 and July 2015.

## 2. Results and Discussion

Parasites of marine mammals encompass species that may cause serious pathological lesions and have been included among possible factors of cetacean stranding [16,17,18]. Moreover, the evaluation of the effects of parasites on these animals is considered essential for planning cetacean management and conservation measures [19]. Therefore, the knowledge of the cetacean parasitic fauna in a specific geographical area may contribute not only to the acquisition of new information on pathogens of these animals but also to possible tools for parasite control in the area. To date, data about parasite infections of cetaceans stranded along the Tuscan coastline of the “Pelagos Sanctuary” (central Italy) are still scarce [19].

Results obtained in this study showed a high prevalence of parasite infections in dolphins stranded along the coasts of Tuscany in the considered period. Twenty one out of 26 examined dolphins tested positive for parasites, with an overall prevalence of 80.77% (21/26). More specifically, 13/15 (86.67%) striped dolphins, 7/10 (70.00%) bottlenose dolphins, and the single Risso’s dolphin were found positive, but no statistical differences emerged among the different prevalence values found in striped dolphins and bottlenose dolphins (*p* = 0.390). In Table 1 are summarized data regarding identified parasite species, their prevalence, and the respective 95% confidence intervals observed in examined striped and bottlenose dolphins.

Overall, all parasite species identified in this study were already reported in previous studies both in dolphins living in the Mediterranean Sea and in other seas [12,20,21,22,23].

**Figure 1:**
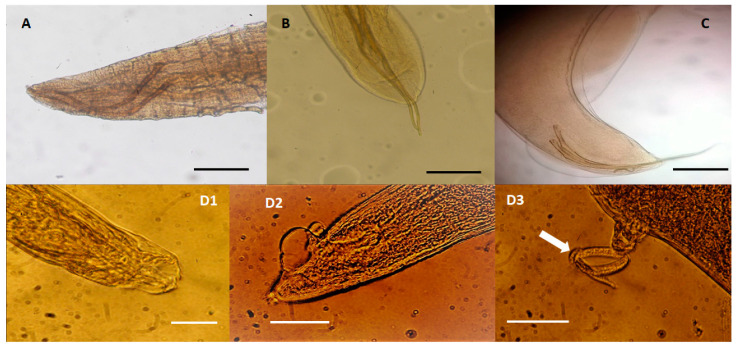
Nematode species identified in dolphins (15 *Stenella coeruleoalba* and 10 *Tursiops truncatus*), stranded along the Tuscan coastline (central Italy) of the “Pelagos Sanctuary” in the period between February 2013 and July 2015. (**A**) *Halocercus lagenorhynchi* adult male, measuring about 7 cm in length and 0.38 mm in width, found in the bronchi of a striped dolphin (*S. coeruleoalba*). Caudal end showing the spicules of about 0.65 mm in length and a copulatory bursa indistinguishable from the cuticle, scale bar 250 µm; (**B**) *Halocercus delphini* adult male measuring about 8 cm in length and 0.46 mm in width, found in the bronchi of a striped dolphin (*S. coeruleoalba*). Caudal end showing the spicules of about 0.73 mm in length, scale bar 250 µm; (**C**) *Skrjabinalius guevarai* adult male, measuring 6 cm in length and 0.5 mm in width, found in the bronchi of a bottlenose dolphin (*T. truncatus*): caudal end showing the spicules (length 0.77–0.80 mm), scale bar 250 µm; (**D1**–**D3**) *Stenurus ovatus* specimens found in the bronchi of a bottlenose dolphin (*T. truncatus*). (**D1**) Caudal end of and adult male with a caudal bursa showing two lateral rays (about 0.0465 mm in length and 0.020 mm wide) and a dorsal ray 0.053 mm long and 0.017 mm wide, (scale bar 250 µm); (**D2**) Caudal end of adult female showing two vulvar lips, one anterior long 0.035 mm and one posterior of about 0.037 mm in length, (scale bar 250 µm). (**D3**) First stage larva of 0.26 mm in length (arrow, scale bar 250 µm).

Among nematodes (Table 1, Figure 1), different respiratory species were identified in striped and bottlenose dolphins examined in this study, including *Halocercus lagenorhynchi* (6.67%, 1/15; Figure 1A) and *Halocercus delphini* (13.33%, 2/15; Figure 1B) that were identified in striped dolphins (Table 1), while *Skrjabinalius guevarai* (20.0%, 2/10; Figure 1C) and *Stenurus ovatus* (20.00%, 2/10; Figure 1D1–D3) were identified in bottlenose dolphins (Table 1). However, while the prevalence of *S. guevarai* in bottlenose dolphins from this study is similar to that (23.0%) reported by Manfredi et al. [24], the prevalence of *S. ovatus* is much higher than that (8.6%) reported by Manfredi et al. [24]. *H. lagenorhynchi* and *H. delphini* were identified in striped dolphins with a prevalence (respectively, 6.67% and 13.33%) higher than that (6.0%) previously reported in Italy [25]. The pathogenic effects of these nematodes depend on the species involved, the intensity of the infection, and on some factors related to the host. Species infecting the lung parenchyma, such as *H. lagenorhynchi* and *S. guevarai,* are a potential cause of death consequent to pneumonia and total or partial occlusion of bronchi and bronchioles and of reduced immersion capacity of infected animals [24,26].

In this study, subcutaneous nematodes belonging to the genus *Crassicauda* (7.67%, 2/26) were detected in a striped dolphin (6.67%, 1/15) and in a bottlenose dolphin (10.00%, 1/10), but their identification at the species level was not possible due to the poor state of parasite conservation. In Italy, the prevalence of *Crassicauda* spp. nematodes is about 15%, and common, striped, bottlenose, and Risso’s dolphins have been found frequently infected [24,25]. The life cycle of these nematodes is still unknown, but cetaceans act as definitive hosts [27].

Although frequently reported in dolphins [28,29], in this study the detection of anisakid nematodes was not possible, probably due to the lack of availability for parasitological examination of the stomach content of examined dolphins.

**Figure 2:**
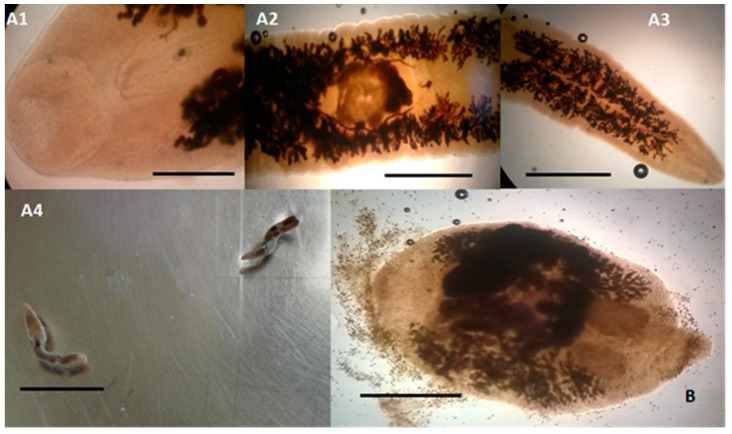
Trematodes identified in dolphins (15 *Stenella coeruleoalba* and 10 *Tursiops truncatus*), stranded along the Tuscan coastline (central Italy) of the “Pelagos Sanctuary” in the period between February 2013 and July 2015. (**A1**–**A4**) *Campula palliata* adult specimens found in the bile ducts of a striped dolphin (*S. coeruleoalba*). (**A1**) Microscopical view of the anterior end showing the oral sucker (scale bar 300 µm); (**A2**) microscopical view of the middle part of the body showing the ventral sucker (scale bar 1 mm); (**A3**) microscopical view of the posterior end of the body (scale bar 1 mm); (**A4**) microscopical view of the entire body of two adult specimens of 12–13 mm in length and 1.7–2.0 mm in width (scale bar 12 mm). (**B**) *Pholeter gastrophilus* adult (2.90 mm long and 2.00 mm wide) found in the submucosa of the third gastric compartment of a bottlenose dolphin (*T. truncatus*) showing a spindle-shaped body, with a cuticle covered with small pointed spines. The uterus, long and folded, is placed marginally and follows the body for almost its entire length. The testicles are ovoid in shape and are placed side by side in the posterior region of the body, while the lobed ovary is placed slightly in front and laterally the testicles, (scale bar 1 mm).

Among flukes (Table 1, Figure 2), the species *Campula palliata* (Figure 2A1–A4) was identified in 3.85% (1/26) of examined dolphins, precisely a striped dolphin, compared to 12.93% previously observed in Italy [24]. *C. palliata* infects the liver parenchyma and liver and pancreatic ducts, and it is often responsible for granulomatous hepatitis leading to decreased liver function, liver failure, and secondary bacterial infections [30]. The species *Pholeter gastrophilus* (Figure 2B) showed an overall prevalence of 26.92% (7/26). However, while the prevalence of this trematode observed in this study in bottlenose dolphins (30.00%, 3/10) was similar to that reported in previous studies in this cetacean species [21,31], in the striped dolphins (26.67%, 4/15) here examined it was lower than that reported in the study of Aznar et al. [22]. The localization site of *P. gastrophilus* is the gastric submucosa, and it is a common cause of granulomatous gastritis, which hinders the food transit and compromise the stomach motility [32]. How odontocetes, the definitive hosts, acquire *P. gastrophilus* and *C. palliata* infection it is not yet known, but it is thought to occur with the ingestion of infected fish and cephalopods, intermediate hosts [11,33].

**Figure 3:**
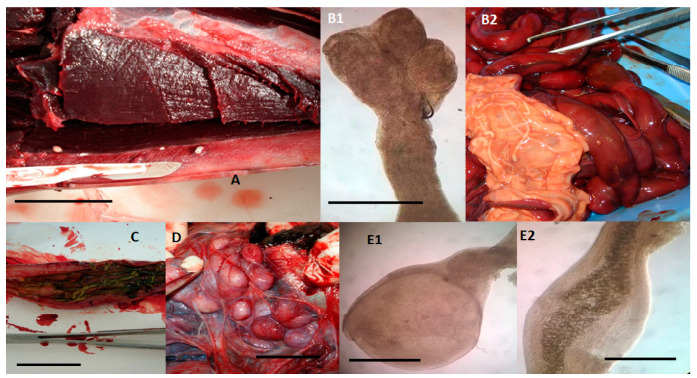
Cestodes and Acanthocephalans identified in dolphins (15 *Stenella coeruleoalba*, 10 *Tursiops truncatus*, and a *Grampus griseus*), stranded along the Tuscan coastline (central Italy) of the “Pelagos Sanctuary” in the period between February 2013 and July 2015. (**A**) *Phyllobothrium delphini* located in the subcutaneous adipose tissue of the perigenital region of a striped dolphin (*S. coeruleoalba*), macroscopic view. Merocercoid larvae appear as white oval cystic formations with a diameter of 5–10 mm and containing an invaginated scolex showing four grooves (bothria) and a short neck, scale bar 2 cm. (**B1**,**B2**) *Tetrabothrium forsteri* adults in the intestine of a striped dolphin (*S. coeruleoalba*), microscopical view of the scolex showing four bothria measuring 0.3–0.69 mm in length and 0.25–0.6 mm in width (**B1**, scale bar 1 mm), and macroscopic view of the strobila whose length may range from a few millimetres to two metres, while the proglottids are wider than long (**B2**). (**C**) *Strobilocephalus triangularis* adults in the intestine of a striped dolphin (*S. coeruleoalba*). The size of the strobila varies from a few millimetres to two meters, while the scolexes are 5–6 mm wide and 4–6 mm long with four muscular bothria (scale bar 2.5 cm). (**D**) *Monorygma grimaldii* merocercoids in the subserosa of the abdominal cavity of an infected striped dolphin (*S. coeruleoalba*). Merocercoids appear as white cystic formations with a diameter of 10–20 mm, each containing an invaginated scolex showing four bothria and a very long neck, scale bar 1.5 cm. (**E1**,**E2**) *Bolbosoma vasculosum* female adult specimen about 0.435 mm wide and 0.85 mm long, found in the intestine of a bottlenose dolphin (*T. truncatus*) showing the bulbar anterior end of the body (**E1**, scale bar 250 µm) and developed eggs (**E2**, scale bar 250 µm).

Among cestodes (Table 1, Figure 3), the overall prevalence of the species *Phyllobothrium delphini* and *Monorygma grimaldii* was, respectively, 42.30% (11/26) and 23.08% (6/26). However, in striped dolphins, the prevalence of *P. delphini* (73.33%, 4/15) and *M. grimaldii* (33.33%, 5/15) was higher than that reported in previous studies [12,24]. The positivity to *M. grimaldii* was also found in the single examined specimen of Risso’s dolphin. In the life cycle of these parasites, dolphins are second intermediate hosts and contain the second (merocercoid) larval stage, while sharks are the definitive hosts [11,34]. However, it is not yet known how dolphins acquire the infections and which are the first intermediate hosts [11,34]. In dolphins, *P. delphini* infects the subcutaneous adipose tissue (Figure 3A), while *M. grimaldii* can be found in the subserosa of the abdominal cavity (Figure 3D). Compared to *P. delphini*, *M. grimaldii* is more frequently the cause of suppurative inflammations. Nevertheless, it is assumed that the damage to the subcutaneous adipose tissue caused by a high *P. delphini* load may have a serious negative impact on swimming abilities of infected dolphins [34].

Among cestode species for which dolphins are the definitive hosts (Table 1, Figure 3), the species *Tetrabothrium forsteri* (7.69%, 2/26; Figure 3B1,B2) and *Strobilocephalus triangularis* (7.69%, 2/26; Figure 3C) were here identified only in striped dolphins, both with a prevalence of about 13% (2/15). This prevalence is lower than that previously reported [20]. Both species infect the intestine but, while *S. triangularis* may be responsible for granulomatous nodular lesions, *T. forsteri* is considered a low-pathogenic parasite [35]. The life cycle of these cestodes is not completely known, but crustaceans are thought to act as first intermediate hosts, while cephalopods and teleost fishes are considered second intermediate or paratenic hosts, containing the plerocercoid larvae, which are infective for dolphins [11].

About acanthocephalans, *Bolbosoma vasculosum* (7.69%, 2/26; Figure 3E1,E2) was identified in bottlenose dolphins with a prevalence (20.00%, 2/8) lower than that (52.0%) reported by Mateu et al. [20]. These parasites are found in the intestine and are potentially highly pathogenic due to the hooked proboscis, which can cause an intestinal inflammatory reaction with fibrosis [33]. The life cycle of *Bolbosoma* spp. is thought to involve the pelagic marine zooplankton as intermediate host and different species of fish as transport hosts [36,37]. Juvenile forms (cystacanths) of these acanthocephalans are widely considered to be the infective stage for cetacean definitive hosts [37].

Faecal analysis by the sedimentation technique with formol-ethyl acetate allowed the detection of trematode eggs (*P. gastrophilus* and *C. palliata*) in 3/15 striped dolphins (20.0%), while 4/15 striped dolphins faecal samples (26.0%) scored positive for cestode eggs at flotation test. On the contrary, all faecal samples from bottlenose dolphins scored negative.

In recent studies, *Giardia duodenalis* and *Cryptosporidium* spp. infections have been recorded in dolphins and included zoonotic genotypes [38,39]. Nevertheless, in this study, as observed in a previous study [40], all dolphins scored negative to *Giardia* spp. and *Cryptosporidium* spp. All tests performed on faecal samples (fresh and Lugol, modified Ziehl–Neelsen, and DiffQuik stained faecal smears, immunoassay, and PCR) tested negative for *G. duodenalis* and *Cryptosporidium* spp. However, due to the intermittent faecal excretion of these protozoans or to a very small amount of protozoan antigen in the examined samples, false negative results may be possible [41].

*T. gondii* is a worldwide diffused zoonotic parasite affecting humans and animals, showing an heteroxenous lifecycle involving intermediate hosts, virtually all warm-blooded animals, and felid definitive hosts [42,43]. In dolphins, *T. gondii* is considered one of the main causes or a main contributing cause of stranding episodes and mortality [18,44,45,46]. In fact, in dolphins, *T. gondii* infection frequently causes encephalitis, myocarditis, lymphadenitis, abortion, and death [47,48]. Six out of 26 (23.08%) examined dolphins (5 striped dolphins and 1 bottlenose dolphin) tested positive to *T. gondii* antibodies at serology, but among them only the brain tissue of a single dolphin was positive for *Toxoplasma* DNA by PCR. In particular, the subject tested PCR positive was a striped dolphin specimen showing a serological positive titre of 1/2560. In previous studies [19,23,48], the seroprevalence of *T. gondii* in dolphins stranded along the Italian coasts was found to vary from 11% to 93%. Therefore, the serological prevalence observed in this study falls within the seroprevalence range reported in Italy. In some investigations [46,49], *T. gondii* serological prevalence was found to be higher in the coastal dolphin species, such as the bottlenose dolphin. Nevertheless, in this study the seroprevalence of *T. gondii* was higher in striped dolphins (33.33%, 5/15), a pelagic species, than in bottlenose dolphins (10.00%, 1/10). Differences between serological and PCR results can be explained by the heterogeneous distribution of *T. gondii* and parasite absence in the low amount of nervous tissue (few mgs) used for molecular analysis, especially in the case of low infection intensity [50]. It is interesting to note that the only subject found PCR positive in this study showed the highest serological titre. Nevertheless, PCR negativity to *T. gondii* of nervous tissues from serologically positive dolphins has been previously reported [19,50]. Furthermore, it is also possible that the positivity observed in some animals in this study was due to infections caused by other protozoa antigenically related to *T. gondii* and reported in dolphins, such as *Neospora caninum* and *Sarcocystis* spp. [51,52], or to other false positive results due to the low specificity of the serological test used [53]. However, this serological test is one of the most used and considered valid for the diagnosis of *T. gondii* [54].

Results of multilocus RFLP-PCR genotyping of *T. gondii*, using genes described by Su and colleagues [55,56], identified the amplified sample as belonging to Type II genotype, excluding for SAG1 gene where type II and III are indistinguishable (Table 2, Figure S1). Although some atypical genotypes were previously identified in dolphins [57,58], the type II genotype is the most widespread genotype in marine mammals in North America and Europe [57,58].

How dolphins may acquire *T. gondii* infection is still controversial, although the ingestion of contaminated water or fish are currently considered the most likely route [44,47,52]. It has been suggested that oocysts poured into the sea with surface waters and sewers containing faeces of the final felid hosts may contaminate the marine environment with oocysts [11]. Oocysts are in fact very resistant in the marine environment, as they can reach infectivity and remain alive and infectious for several months [59]. It is also possible that the discharges of boats may play an important role in pelagic environments, especially if cats are on board [46]. However, it is known that dolphins ingest modest quantities of water, deriving mainly from the diet, and that they feed mainly on fish, cephalopods, and crustaceans that are not intermediate hosts of *T. gondii* [60]. On the other hand, some recent studies have demonstrated that fish and bivalve molluscs may contain viable *T. gondii* oocysts and can be involved in the transmission of *T. gondii* in the marine environment [47,61]. Bivalve molluscs are not usually part of the dolphin diet; however, the actual role of fish in the infection of dolphins has also not yet been clarified.

## 3. Materials and Methods

### 3.1. Animals

This study was carried out in the period between February 2013 and July 2015 and involved 26 dolphins found stranded lifeless in an optimal state of preservation on the Tuscan coastline (central Italy) of the Pelagos Sanctuary and promptly submitted for necropsy. Collection and transport of all carcasses were performed according to the EC Regulation 1069/2009. In this period, the following cetaceans were examined: 15 striped dolphins (*S. coeruleoalba*), 10 bottlenose dolphins (*T. truncatus*), 1 Risso’s dolphin (*G. griseus*). Biological data of these animals and geographic localities where they were found are presented in Table 3.

### 3.2. Parasitological Examination

All dolphins were necropsied and all organs, including the subcutaneous adipose tissue, lung, liver, stomach, and intestine were examined by using different parasitological techniques aimed to the collection and identification of parasites. However, for most of examined specimens, the stomach content was not fully available for parasitological examination, as it was used for the identification of food composition and feeding habits of dolphins.

After collection, helminths were fixed and preserved in 70% ethanol. When necessary, worms were cleared with lactophenol or glycerine. After clarification, internal and external structures were visualized under an optical microscope and measured by using a micrometric ocular. Parasite identification was based on morphological identification keys available in the literature [11,12,20,23,30,32,35,37,62,63,64,65,66,67].

Moreover, individual faecal samples were collected from all examined dolphins and promptly analysed or stored at 4 °C and examined within 24 h. All samples were analysed macroscopically for macroparasites, such as nematode (sub)adults, proglottids of cestodes, and worm fragments, and then screened microscopically by the flotation test with a low-density solution (33% ZnSO_4_ solution) and by using the formalin-ethyl acetate centrifugation technique [39,68]. Fresh and Lugol, modified Ziehl–Neelsen, and Diff Quick stained faecal smears were performed for the search of protozoa, especially *Giardia* spp. and *Cryptosporidium* spp. [39,68]. In addition, a commercial rapid immunoassay (RIDA QUICK *Cryptosporidium*/*Giardia* Combi, R-Biopharm, Darmstadt, Germany) was used on the same faecal samples for the search of *G. duodenalis* and *Cryptosporidium* spp. faecal antigens.

PCR analysis was also performed for *Giardia* spp. and *Cryptosporidium* spp. on faecal samples as described by Cacciò et al. [69] and Pedraza-Diaz et al. [70], respectively. The extraction of DNA from stool was carried out by using a QIAamp DNA stool mini kit (Qiagen, Milan, Italy), following the instructions of the manufacturer.

During necropsy, blood clots were collected from the heart chamber of each dolphin and centrifuged (at 2500 rpm for 10 min). Obtained serum samples were screened for anti-*Toxoplasma gondii* antibodies by using a commercial direct agglutination test (Toxo-Screen DAT, BioMérieux, Florence, Italy) accordingly to manufacturer’s instructions. IgM-mediated agglutination was suppressed by using a diluting buffer containing 2-mercaptoethanol. The test was performed in microtitration plates with U-shaped wells. Control and diagnostic sera were diluted from 1:20 to 1:5200. The minimal titre considered as positive was greater or equal to 1:20, as reported for marine mammals [57,71,72,73].

Brain tissue samples were used for molecular analysis aimed to detect *T. gondii* DNA as described by [74]. The extraction of DNA from brain tissues was carried out by using a DNeasy Blood and Tissue Kit (Qiagen, Milan, Italy), following the manufacturer’s instructions. Positive DNA samples were obtained using BigDye^®^ Terminator v1.1 Cycle Sequencing Kit (Applied Biosystems, Life Technologies, Thermo Fisher Scientific, Milan, Italy), purified using BigDye X terminator^®^ Purification Kit (Applied Biosystems, Thermo Fisher Scientific, Milan, Italy), and sequenced with the 310 Genetic Analyser (Applied Biosystems, Thermo Fisher Scientific, Milan, Italy). The samples confirmed as *T. gondii* positive were genotyped through multilocus PCR-RFLP genotyping by using the genetic markers described by Su and colleagues [55,56].

### 3.3. Statistical Analyses

The prevalence of parasites and the corresponding 95% confidence intervals (95% CIs) were calculated. Results were statistically analysed in order to evaluate possible differences between data found in the two most represented dolphin species in this study (striped dolphin and bottlenose dolphin). Data were analysed using a χ2 test with the Yates correction or a Fisher test [75], when appropriate. Results were considered significant when the null hypothesis had a probability lower than *p* < 0.05.

## 4. Conclusions

Results from this study showed a high prevalence of parasite infections in dolphins stranded along the coasts of Tuscany. For some parasite species, observed prevalence was much higher than that previously reported. The high prevalence of endoparasite infections in the subjects herein examined and the identification of parasite species considered as a cause of severe debilitation or death highlight the importance of parasite monitoring in investigations aimed at evaluating the health status of dolphins in the Pelagos Sanctuary, especially for threatened cetacean species [76]. Moreover, the life cycle of many identified parasite species and, mainly, the routes of infection in dolphins are still poorly understood, and further studies are needed to fill these gaps.

## Figures and Tables

**Table 1:** Prevalence and corresponding 95% confidence intervals (95% ICs) of parasites identified in striped dolphins and bottlenose dolphins found stranded between February 2013 and July 2015 along the coastline of Tuscany (Pelagos Sanctuary, central Italy).

Parasites	Positive	Negative	Prevalence	IC95 %
**Striped dolphin (** ***Stenella coeruleoalba*** **, *n* = 15)**				
**Nematodes**				
*Halocercus lagenorhynchi*	1	14	6.67%	0.00–19.29
*Halocercus delphini*	2	13	13.33%	0.00–30.54
*Crassicauda* spp.	1	14	6.67%	0.00–19.29
**Trematodes**				
*Pholeter gastrophilus*	4	11	26.67%	4.29–49.05
*Campula palliata*	1	14	6.67%	0.00–19.29
**Cestodes**				
*Phyllobothrium delphini*	11	4	73.33%	50.95–95.71
*Monorygma grimaldii*	5	10	33.33%	9.48–57.19
*Tetrabothrium forsteri*	2	13	13.33%	0.00–30.54
*Strobilocephalus triangularis*	2	13	13.33%	0.00–30.54
**Protozoa (serology)**				
*Toxoplasma gondii*	5	10	33.33%	9.48–57.19
**Bottlenose dolphin (** ***Tursiops truncatus*, *n* = 10)**				
**Nematodes**				
*Skrjabinalius guevarai*	2	8	20.00%	0.00–44.79
*Stenurus ovatus*	2	8	20.00%	0.00–44.79
*Crassicauda* spp.	1	9	10.00%	0.00–28.59
**Trematodes**				
*Pholeter gastrophilus*	3	7	30.00%	1.60–58.40
**Acanthocephalans**				
*Bolbosoma vasculosum*	2	8	20.00%	0.00–44.79
**Protozoa (serology)**				
*Toxoplasma gondii*	1	9	10.00%	0.00–28.59

**Table 2:** Genotyping of *Toxoplasma gondii* isolate from a striped dolphin (*Stenella coeruleoalba*).

Genetic Markers						
	SAG1 *	5′ +3′ SAG2 **	alt SAG2 ***	SAG3	BTUB	GRA6
Genotype	II/III	II	II	II	II	II

* At SAG1 locus, type II and III are indistinguishable; ** SAG2 marker based on 5k and 3k ends of the gene sequence; *** an SAG2 marker based on the 5k end of the gene sequence but different from 5k-SAG2 [55,56].

**Table 3:** Species, gender, and weight of examined cetaceans found stranded between February 2013 and July 2015 along the coastline of Tuscany (Pelagos Sanctuary, central Italy) and year and geographic localities where animals were found.

Species	Gender	Weight (kg)	Year of Stranding	Geographic Localities (Geographic Coordinates)
*Stenella coeruleoalba*	M	49	2013	Tirrenia (Pisa) 43°37′38″ N–10°17′28″ E
*Stenella coeruleoalba*	M	100	2013	Monte Argentario (Grosseto) 42°26′07″ N–11°07′00″ E
*Stenella coeruleoalba*	M	-	2013	Orbetello (Grosseto) 42°26′22″ N–11°12′45″ E
*Stenella coeruleoalba*	F	45	2013	Follonica (Grosseto) 42°55′08″ N–10°45′41″ E
*Stenella coeruleoalba*	F	61	2013	Calambrone (Pisa) 43°35′49″ N–10°17′40″ E
*Stenella coeruleoalba*	M	65	2013	Piombino (Livorno) 42°56′05.37″ N–10°31′19.63″ E
*Stenella coeruleoalba*	F	25	2013	Bibbona (Livorno) 16′12.64″ N–10°35′54.73″ E
*Stenella coeruleoalba*	M	50	2013	Livorno 43°32′36″ N–10°19′1″ E
*Stenella coeruleoalba*	M	6	2013	Livorno 43°32’36” N–10°19′1″ E
*Stenella coeruleoalba*	M	75	2013	Marina di Grosseto (Grosseto) 42°42′55″ N–10°59′02″ E
*Stenella coeruleoalba*	F	60	2013	Orbetello (Grosseto) 42°26′22″ N–11°12′45″ E
*Stenella coeruleoalba*	M	75	2014	Cecina (Livorno) 43°18′42.53″ N–10°31′08.49″ E
*Stenella coeruleoalba*	M	-	2014	Piombino (Livorno) 42°56′05.37″ N–10°31′19.63″ E
*Stenella coeruleoalba*	M	29.5	2015	Portoferraio (Livorno) 42°48′45″ N–10°18′56″ E
*Stenella coeruleoalba*	M	45	2015	Marina di Massa (Massa) 44°00′33.8″ N–10°06′06.9″ E
*Tursiops truncatus*	M	-	2013	Marina di Castagneto Carducci (Livorno) 43°10′39.93″ N–10°32′20.27″ E
*Tursiops truncatus*	*	-	2013	Calambrone (Pisa) 43°35′49″N–10°17′40″E
*Tursiops truncatus*	M	140	2013	Calambrone (Pisa) 43°35′49″N–10°17′40″E
*Tursiops truncatus*	F	70	2013	Rosignano Marittimo (Livorno) 43°24′32″ N–10°28′26″ E
*Tursiops truncatus*	F	27	2014	Livorno 43°32′36″ N–10°19′1″ E
*Tursiops truncatus*	M	300	2014	Marina di Pietrasanta (Lucca) 43°55′35.1″ N–10°11′48.3″ E
*Tursiops truncatus*	M	30	2014	Tirrenia (Pisa) 43°37′38″ N–10°17′28″ E
*Tursiops truncatus*	M	140	2014	Rosignano Marittimo (Livorno) 43°24′32″ N–10°28′26″ E
*Tursiops truncatus*	F	102	2015	Marina di Pisa (Pisa) 43°40′20″ N–10°16′37″ E
*Tursiops truncatus*	F	43	2015	Livorno 43°32′36″ N–10°19′1″ E
*Grampus griseus*	F	-	2015	San Vincenzo (Livorno) 43°06′02.28″ N–10°32′11.96″ E

-: Data not available; *: sex was not identified.

## Supplementary Materials

The following are available online at https://www.mdpi.com/2076-0817/9/8/612/s1, Figure S1: Multiplex multilocus nested PCR-RFLP (Mn-PCR-RFLP) analysis of *Toxoplasma gondii* sample using seven different genetic markers. Nested PCR products from each marker were digested with selected restriction enzymes [56,57], and DNA fragments were separated in agarose.
