# Supplementary material for: Dolphins Stranded along the Tuscan Coastline (Central Italy) of the “Pelagos Sanctuary”: A Parasitological Investigation
Source: Pathogens. 2020 Jul 27;9(8):612. doi: 10.3390/pathogens9080612 (PMC7459703; doi:10.3390/pathogens9080612)
Supplement: Supplementary file 1 [file pathogens-09-00612-s001.pdf]

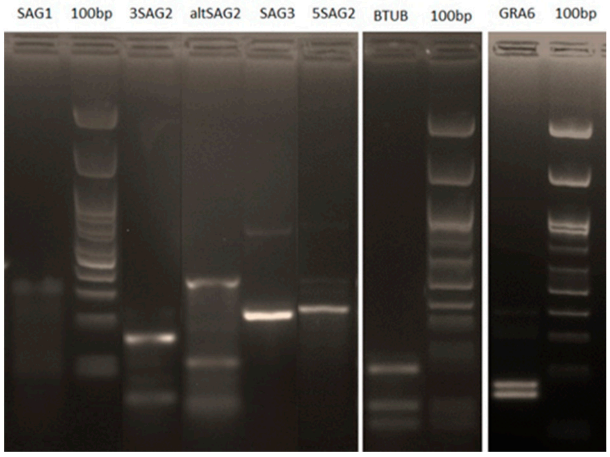

Figure S1. Multiplex multilocus nested PCR-RFLP (Mn-PCR-RFLP) analysis of *Toxoplasma gondii* sample using seven different genetic markers. Nested PCR products from each marker were digested with selected restriction enzymes [56,57], and DNA fragments were separated in agarose.
